# A cross-sectional cohort study of the activity and turnover of neutrophil granulocytes in juvenile idiopathic arthritis

**DOI:** 10.1186/s12969-021-00600-7

**Published:** 2021-06-30

**Authors:** Malin Backlund, Per Venge, Lillemor Berntson

**Affiliations:** 1 Department of Women’s and Children’s Health, Uppsala University, Uppsala, Sweden; 2 Department of Medical Sciences, Uppsala University, Uppsala, Sweden

**Keywords:** Neutrophil granulocytes, Priming, Granule proteins, Juvenile idiopathic arthritis

## Abstract

**Background:**

The inflammatory process in juvenile idiopathic arthritis (JIA) involves both the innate and the adaptive immune system. The turnover and activity of neutrophil granulocytes may be reflected by proteins secreted from primary or secondary granules and from the cytoplasm of sequestered cells. Our primary aim was to compare the levels of the secondary neutrophil granule protein human neutrophil lipocalin (HNL), in JIA patients and controls, and to explore a possible priming of neutrophils through parallel analyses in plasma and serum. A secondary aim was to relate the levels of HNL to two other well-studied leukocyte proteins, S100A8/A9 and myeloperoxidase (MPO), as well as to clinical aspects of JIA.

**Methods:**

The concentrations of the three biomarkers in serum, two of them also in plasma, were measured using enzyme-linked immunosorbent assay in 37 children with JIA without medical treatment, in high disease activity based on juvenile arthritis disease activity score 27 (JADAS27), 32 children on medical treatment, mainly in lower disease activity, and 16 healthy children. We assessed for differences between two groups using the Mann-Whitney U test, and used the Kruskal-Wallis test for multiple group comparisons. Spearman rank correlation, linear and multiple regression analyses were used for evaluation of associations between biomarker concentrations and clinical scores.

**Results:**

The concentrations of HNL and MPO in serum were significantly increased in children with JIA (*p* < 0.001, *p* = 0.002) compared with healthy children, but we found no difference in the plasma levels of HNL and MPO between children with JIA and controls. The serum concentrations of MPO and HNL were unaffected by medical treatment, but S100A8/A9 was reduced by medical treatment and correlated with JADAS27 in both univariate (r = 0.58, *p* < 0.001) and multivariate (r = 0.59, p < 0.001) analyses.

**Conclusions:**

Neutrophil granulocytes in children with JIA are primed to release primary and secondary granule proteins, without relation to medical treatment, whereas signs of increased turnover and sequestration of neutrophil granulocytes are reduced by treatment. Levels of neutrophil-originating proteins in serum most likely reflect underlying disease activities of JIA.

**Supplementary Information:**

The online version contains supplementary material available at 10.1186/s12969-021-00600-7.

## Background

Juvenile idiopathic arthritis (JIA) is the most common rheumatic condition in childhood. The term encompasses seven disease categories, classified based on the International League of Associations for Rheumatology criteria [1].

The cause of the disease is multifactorial. JIA was previously thought to originate from the adaptive immune system [2, 3], but there is growing evidence that its pathophysiology also involves the innate immune system [4, 5]. The neutrophil granulocyte, the most abundant type of white blood cell, is part of the innate immune system. It is the first immune cell recruited when an inflammatory response is initiated. For a long time, it was believed to have a short lifespan when activated. However, new studies have shown that the neutrophil can avoid apoptosis under inflammatory conditions, thereby extending its lifespan and opening for a more prominent role in inflammatory disease [6].

Studies have found the neutrophil concentration to be high in the joint fluid of JIA patients [7]. Transcriptional expression of neutrophil granule protein genes has been shown to be increased in JIA peripheral blood mononuclear cells [8] and neutrophils in JIA exhibit considerable specificity in their transcriptomes [9]. The relevance of neutrophils in the pathophysiology of JIA is further strengthened by the correlation between S100A8/A9 and disease activity [10, 11]. The protein S100A8/A9, also called calprotectin and myeloid-related proteins 8 and 14 heterocomplex, occurs primarily in the cytoplasm of neutrophil granulocytes and monocytes and is not expressed by lymphocytes [12]. It accounts for 45% of the cytoplasmatic proteins in neutrophils [13].

The dimeric form of human neutrophil lipocalin (HNL; neutrophil gelatinase-associated lipocalin) is a protein expressed exclusively by neutrophils, while the monomeric form of the protein is expressed by both neutrophils and epithelial cells [14]. In the circulation, part of HNL is bound to matrix metalloproteinase-9 (MMP-9), forming a heterodimer [15]. Matrix metalloproteinases are a family of endopeptidases that play essential roles in a wide range of proteolytic processes [16]. Earlier research has shown increased concentrations of MMP-9 in serum and synovial fluid in children with JIA [17].

The protein myeloperoxidase (MPO), secreted from primary granules in neutrophils, has powerful oxidative effects, creating a strong microbicidal property [8]. However, MPO is also regarded as pathogenic in an inflammatory setting, as it promotes inflammation and causes tissue damage [9]. It is expressed in neutrophils and monocytes and has been shown to be increased in JIA [18].

A role of neutrophils in JIA is likely, but remains unclear. As outlined above, several of the inflammatory proteins previously studied in the circulation of JIA patients might originate from neutrophils, but could also come from other cell types. The neutrophil has a lower threshold to release HNL when it has been “preactivated” or “primed.” The primary aim of this study was to investigate levels of neutrophil-specific HNL in serum and plasma as a possible sign of priming of neutrophils. To verify that the enzyme-linked immunosorbent assay (ELISA) method used to measure neutrophil-specific HNL gave a representative concentration of all forms of HNL in the circulation, a western blot analysis was performed. Since one of the dimeric forms of HNL occurs in complex with MMP-9, we also wanted to explore levels of MMP-9.

A secondary aim was to relate levels of HNL to levels of well-known neutrophil proteins, in the two groups of children with JIA, on or off medical treatment, as well as to healthy children. The three neutrophil proteins measured in this report, represent different aspects of neutrophil activity in the circulation of children with JIA: HNL, representing secretion from secondary granules, S100A8/A9, representing sequestration and turnover of neutrophils and MPO representing secretion from primary granules.

## Methods

### Patients

Sixty-nine children diagnosed and classified with JIA based on the International League of Associations for Rheumatology criteria [1], were included in this study. In all, 37 participants were on no medical treatment and 32 were on either methotrexate, a biological agent, or the combination of a biological agent and methotrexate, Table 1. The systemic category was excluded because it, in many aspects, is considered to be an autoinflammatory disease [19]. The polyarticular rheumatoid factor positive category was excluded because of a higher degree of neutrophil extracellular trap formation, which could impact on the measures used in this study [20]. Most patients in the first group were newly diagnosed or had been off medication for at least half a year and had been admitted because of a disease flare and were at higher disease activity. We chose not to categorize disease activity in each participant by JIA category, but rather to study two groups of children with JIA, selected to be as similar as possible: one with children in an untreated state, at a higher disease activity, and one with children on medical treatment at a lower disease activity state. Gender, age at onset, and JIA categories were roughly similar in the two groups of patients, Table 1. We also included 16 healthy children.

**Table 1:** Demographic data in 69 patients with juvenile idiopathic arthritis

	Patients on no medical treatment *n* = 37	Patients on medical treatment *n* = 32	Healthy children *n* = 16
Female sex, n (%)	25 (68)	22 (69)	2 (12)
Age at disease onset, years, median (IQR)	6.8 (2.7–12.6)	4.0 (2.6–11.4)	
Age at sampling, years, median (IQR)	9.9 (5.4–15.2)	12.8 (9.6–14.6)	5.3, (2.0–9.5)**
JADAS27 score	8.6 (5.8–12.8) (*n* = 34)	4.3 (1.3–8.1) (*n* = 29)*	
**JIA categories, course type**	Number	Number	
Oligoarticular persistent	18	12	
Oligoarticular extended	3	3	
Enthesitis-related arthritis	6	1	
Polyarticular RF negative	6	8	
Juvenile psoriatic arthritis	3	3	
Undifferentiated arthritis	1	5	
**Medical treatment**		Number	
Methotrexate		14	
Biological agent		7	
Biological agent + methotrexate		11	

*IQR* interquartile range; *JADAS27* juvenile arthritis disease activity score 27; *RF* rheumatoid factor

*Mann-Whitney U, *p* = 0.002

**Mann-Whitney U, *p* = 0.001

### Healthy children

Samples from controls were collected pre-operatively from children admitted for minor surgery, who were otherwise healthy and on no medication for any disease. Exclusion criteria were also presence of any inflammatory disease, diabetes, any atopic disease with continuous medication or special diet because of intolerance.

### Methods

The study visit included physical examination for joint count (0–27 active joints) and analyses of erythrocyte sedimentation rate, C-reactive protein, leukocytes, and neutrophils. It also included physician’s global assessment of disease activity on a visual analogue scale (VAS) (0–10 cm) and patient-reported global assessment of well-being, assessed by a parent if the child was ≤9 years old (VAS 0–10 cm). The joint count, normalized erythrocyte sedimentation rate ((ESR in mm/h) - 20)/10) to a scale 0–10 [21], physician’s global assessment of disease activity, and patient/parent-reported global assessment of well-being were used for measurement of JADAS27 [22]. Blood samples were centrifuged, aliquoted, and frozen in − 70 °C after a mean of 2.6 h (min–max 1.5–3.8). Analysis of HNL and MPO was performed using ELISA (Diagnostics Development, Uppsala, Sweden); analysis of S100A8/A9 was performed with ImmunoCAP (Phadia AB, Uppsala, Sweden). Inter- and intraassay variations of duplicates were < 10% coefficient of variation for all assays. The levels of MMP-9 were measured using ELISA (Thermo-Fisher Scientific Uppsala, Sweden).

Western blot analysis of HNL and MMP-9 in serum was performed for 6 patients as previously described [14], using polyclonal antibodies against HNL (Diagnostics Development) and monoclonal antibodies against MMP-9.

#### Statistical methods

Conventional descriptive statistics were used and medians and IQRs have been given. The Mann-Whitney U test was used for comparisons of two groups and a chi-squared test was used for comparison of JIA categories between the groups, Table 1. Kruskal-Wallis non-parametric analysis of variance was used for multiple group comparisons and post-hoc analysis (Dunn) was used for pair-wise analyses. Spearman’s rank correlation analyses, linear regression analyses and multiple regression analyses were applied as indicated and used for the evaluation of associations between biomarker concentrations and clinical scores. In the multiple regression analyses, JADAS27 was used as the dependent variable. The variables were removed if *p* > 0.1; thus, only independent variables were included in the model. *P* values of less than 0.05 were considered statistically significant for two-tailed tests. Analyses were carried out using the Statistical Package for Social Sciences version 26 (SPSS Inc., Chicago, IL, USA) and MedCalc® Statistical Software version 19.6 (MedCalc Software Ltd., Ostend, Belgium; https://www.medcalc.org; 2020).

## Results

A total of 69 children with JIA (68.1% girls), median age at sampling 11.8 years, interquartile range (IQR) (6.9–14.9) and 16 healthy children (12.5% girls, median age 5.3 years, IQR 2.0–9.5 years) constituted the study cohort; 37 of the 69 participants were not on medical treatment at inclusion and 32 were, Table 1. JADAS27 was significantly higher among untreated children than among those on medical treatment (*p* = 0.002), but overlapped between the groups. A comparison of age at sampling between the 69 children with JIA and the 16 healthy children showed a *p*-value of 0.001. A chi-squared test used to compare the numbers of untreated and treated children in each category of JIA did not show significance.

**Fig. 1:**
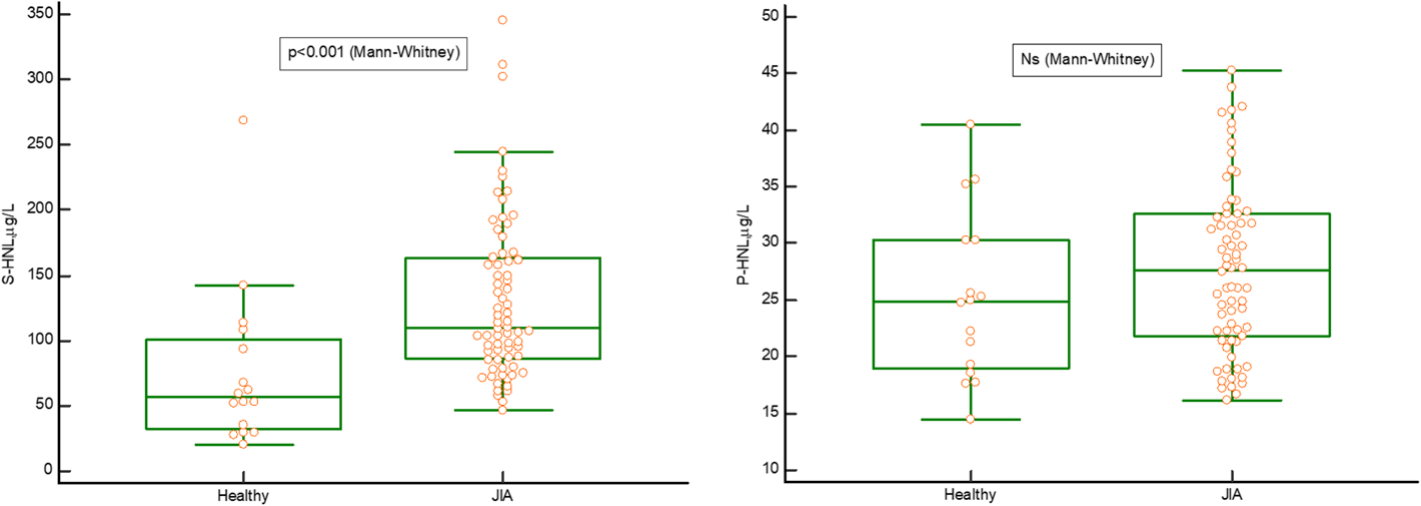
Comparison of serum (**A**) and plasma (**B**) levels of human neutrophil lipocalin (HNL) in 16 healthy children and 69 children with juvenile idiopathic arthritis (JIA). The Mann-Whitney U test was used. Ns. = non-significant

**Fig. 2:**
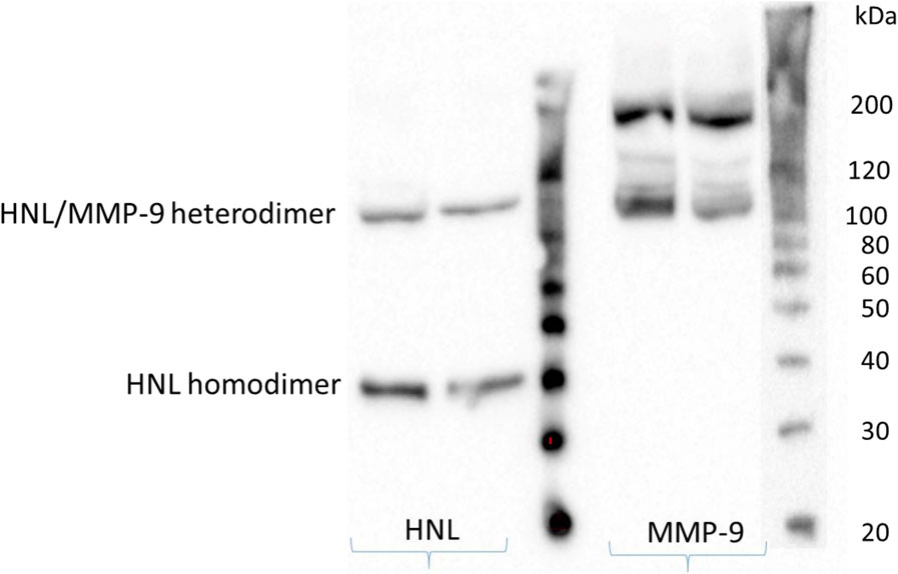
Western blot analysis of human neutrophil lipocalin (HNL) and matrix metalloproteinase-9 (MMP-9) in serum. The result from two patients, among six with identical results, is presented. The left side shows the results of HNL blotting. Only homodimeric and heterodimeric forms of HNL were detected at molecular sizes of approximately 45 kDa and 110 kDa. The right side shows the results of MMP-9 blotting with the lower molecular size of MMP-9 coinciding with the position of HNL at approximately 110 kDa, in addition to a higher molecular band corresponding to approximately 200 kDa

The serum concentration of HNL in the whole group of 69 children with JIA was significantly higher compared with that in healthy children, *p* < 0.001 but the corresponding analysis of HNL in plasma did not show any difference compared with healthy controls, Fig. 1A and B. The serum concentration of MPO in the whole group of children with JIA was significantly higher compared with that in healthy children, *p* = 0.002 (data not shown). The western blot analysis in Fig. 2 shows the existence of homodimeric and heterodimeric HNL in serum from two of six patients with JIA, whereas no monomeric HNL was detected. The results were identical in the six patients. Thus, HNL was detected at molecular weights of 45 and 110 kDa, the latter coinciding with the molecular weight when combined with the smaller form of MMP-9, indicating the existence in serum of homodimeric HNL and HNL in a heterodimeric complex with MMP-9. The larger MMP-9 was likely homodimeric MMP-9. Levels of MMP-9 correlated with levels of HNL in the group of 69 children with JIA, r = 0.8 (data not shown).

**Fig. 3:**
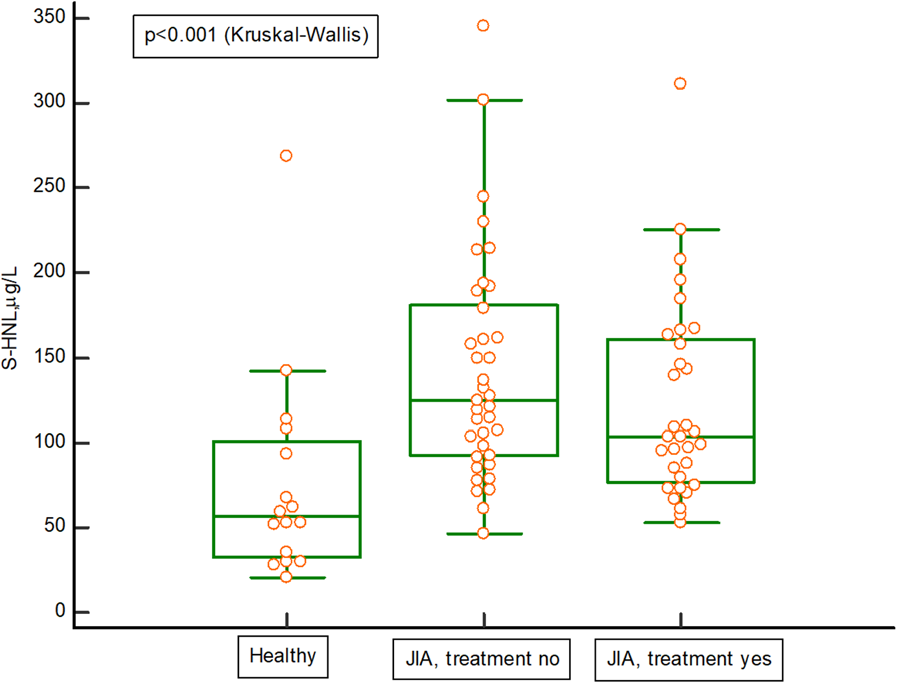
Serum levels of human neutrophil lipocalin (HNL) in 16 healthy children compared with in 69 children with juvenile idiopathic arthritis (JIA). Thirty-seven of the 69 children with JIA were on no medical treatment and 32 of 69 were on treatment. The Kruskal-Wallis test was used, as well as a post-hoc analysis (Dunn): healthy vs. no treatment *p* = 0.001 and healthy vs. treatment *p* < 0.05

**Fig. 4:**
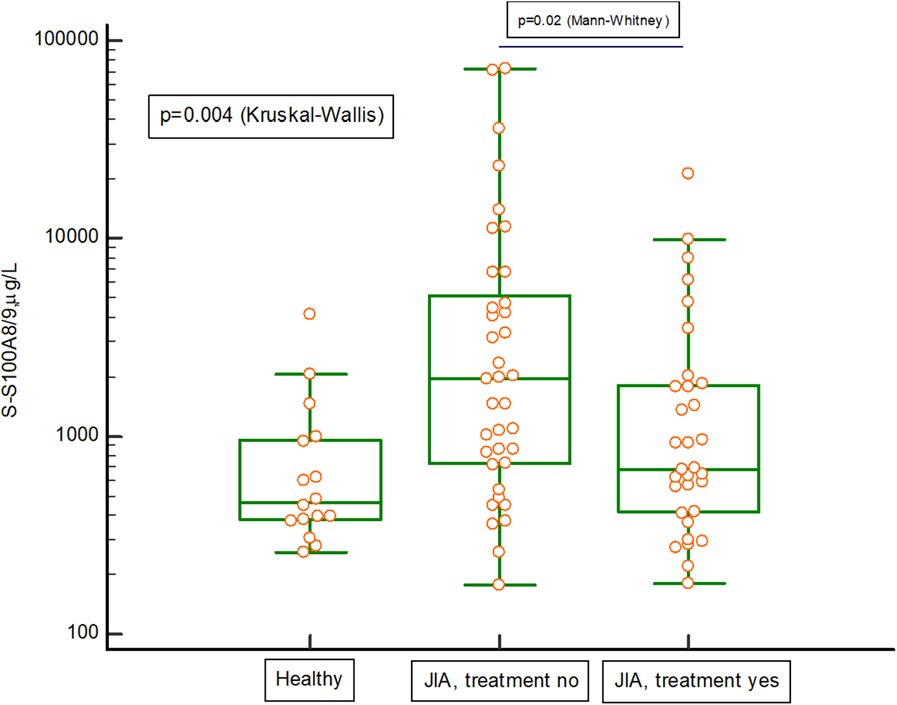
Serum levels of S100A8/A9 in 16 healthy children and 69 children with juvenile idiopathic arthritis (JIA). Thirty-seven of the 69 children with JIA were on no medical treatment, and 32 of 69 were on medical treatment. The Kruskal-Wallis test was used, as well as a post-hoc analysis (Dunn): healthy vs. no treatment *p* = 0.01 and healthy vs. treatment non-significant. Using a Mann-Whitney U test, we also compared the untreated group of children with JIA with the treated group, *p* = 0.02

**Fig. 5:**
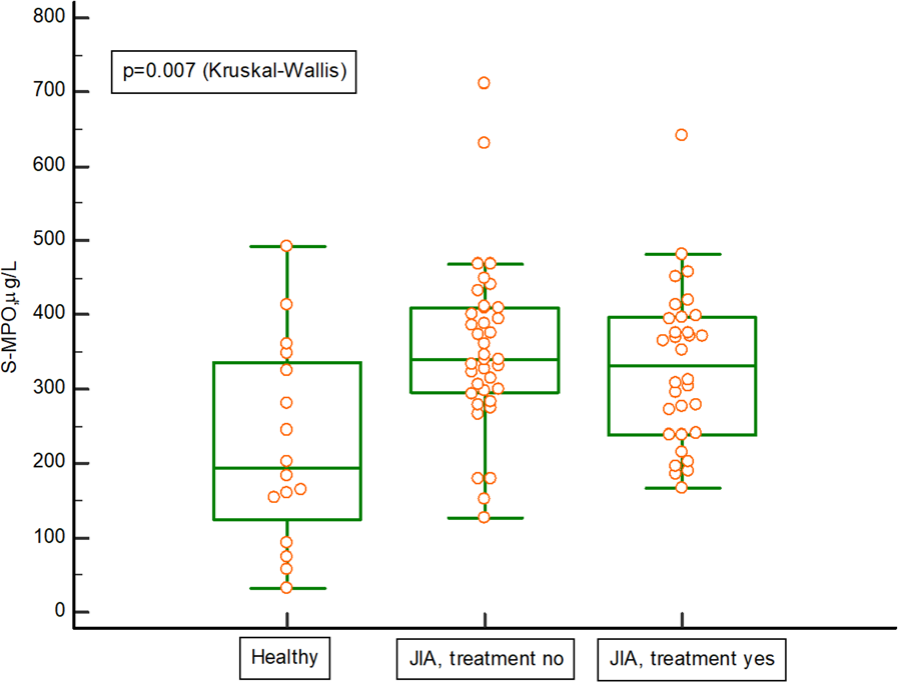
Serum levels of myeloperoxidase (MPO) in 16 healthy children and 69 children with juvenile idiopathic arthritis (JIA). Thirty-seven of the 69 children with JIA were on no medical treatment, and 32 of 69 were on medical treatment. The Kruskal-Wallis test was used, as well as a post-hoc analysis (Dunn): healthy vs. no treatment *p* = 0.01 and healthy vs. treatment *p* < 0.05

The serum concentrations of HNL, S100A8/A9 and MPO, were significantly higher in untreated children with JIA than in healthy children, *p* = 0.001 for HNL (Fig. 3), *p* = 0.01 for S100A8/AA9 (Fig. 4), p = 0.01 for MPO (Fig. 5). The serum concentrations of HNL (Fig. 3) and MPO (Fig. 5), stayed elevated despite medical treatment. Additional Table 1 presents the Spearman’s rank correlation analyses between HNL in serum and levels of B-leukocytes, B-neutrophils, S-C-reactive protein, S-MPO, S-S100A8/A9, S-MMP-9, and JADAS27 in the whole cohort. Additional Figs. 1 and 2 show levels of HNL and S100A8/A9 in the different categories of the disease, in the whole cohort of children with JIA, but the number of children in many categories was very small, hindering the drawing of any conclusions.

**Fig. 6:**
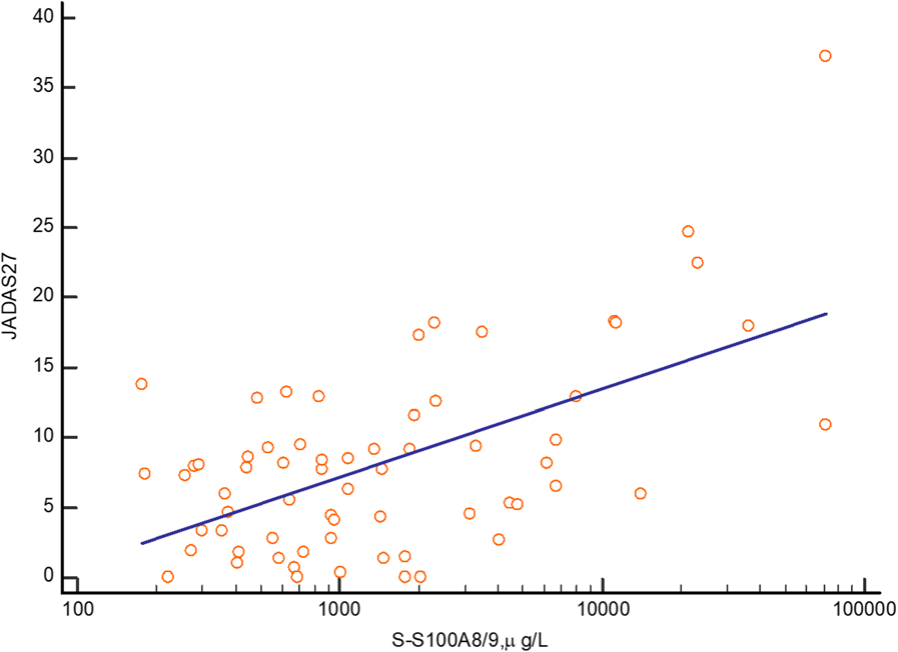
Regression analysis of the relationship between juvenile arthritis disease activity score 27 (JADAS27) and S100A8/A9, measured in the whole cohort of children with JIA, (*n* = 65 in this analysis), r = 0.58, *p* < 0.001

In Fig. 6, a significant positive association between S100A8/A9 and JADAS27 (r = 0.58, *p* < 0.001) in the whole group of children with JIA can be seen. The significant and independent association was also seen after multivariate analysis with JADAS27 as the dependent variable, including S-100A8/A9, S-CRP, P-HNL, S-HNL, P-MPO, S-MPO and B-leukocytes in the model, r = 0.59, *p* < 0.001. The concentrations of HNL in serum showed no association to JADAS27, as seen in Additional Table 1.

## Discussion

In this study of two groups of children with JIA, in different treatment states, we found the serum concentrations of two different proteins from neutrophil granulocytes – HNL, and MPO – to be significantly increased in children with JIA compared with healthy controls. However, plasma concentrations of HNL and MPO were similar to those in healthy controls. There was a significant correlation between the concentration of S100A8/A9 and disease activity, assessed using JADAS27. Concentrations of HNL and MPO did not correlate with disease activity, but were elevated in the group of children on medical treatment.

Human neutrophil lipocalin is stored in secondary granules, the release of which constitutes one of the phenotypic changes that neutrophils undergo when they have been primed. In this study, the western blot analysis supported that the monomeric form of HNL did not exist in significant amounts and that the ELISA method captured mainly the dimeric neutrophil-specific forms of HNL. The concentrations of HNL in serum were significantly higher in JIA patients than in controls, whereas concentrations in plasma were not. This supports the notion of neutrophil priming and illustrates the extent of neutrophil activation, since the serum concentrations are independent of the elimination of HNL occurring in plasma samples [23]. Studies on other autoimmune disorders, such as multiple sclerosis, have also found neutrophils to be characterized by a primed phenotype [24].

To our knowledge, there has been only one other study on the concentration of HNL in JIA. In that study, the MMP-9/HNL complex was increased, irrespective of disease duration, age, or disease category, thus being in line with the results of our study [17]. Those researchers found a weak correlation between the concentration of HNL and the erythrocyte sedimentation rate, as did we (data not shown) – but we also analyzed a possible correlation with JADAS27, with a negative result. Our results of western blot and correlation analyses between levels of MMP-9 and HNL supported there being a complex between HNL and MMP-9.

As far as we know, our study is the first to show an increased concentration of the neutrophil-specific form of HNL, supporting that neutrophils are involved in the inflammatory state of JIA. Apart from being released by neutrophils, HNL can also be released from epithelial cells. Thus, the result of the previous study described above, which found an increased concentration of the MMP-9/HNL complex, might reflect a secretion also from epithelial cells. Increased concentrations of S100A8/A9 do not necessarily represent the involvement of neutrophils, since such proteins are also released from monocytes and could be induced in epithelial cells under inflammatory conditions [12]. Myeloperoxidase is also produced by monocytes [25].

Our results for S100A8/A9 are in line with those of several other studies showing that S100A8/A9 correlates with inflammatory activity in JIA [26]. The highest concentration has been found in the systemic disease category [27]. Though our study did not include any patients with this subtype, the correlation with disease activity was significant also in the multivariate analysis.

One previous study has been conducted on the concentration of MPO in JIA. Studying a small cohort of children, it concluded that medical treatment did not affect MPO concentration [28]. However, studies on rheumatoid arthritis have shown diverse results regarding the association between MPO and disease activity [29, 30], whereas no correlation between HNL and disease activity has been found [31]. Myeloperoxidase is not specific for neutrophils, but occurs in the primary granules of neutrophils. In this study, it was secreted in a similar pattern as HNL, which is not surprising since MPO secretion is increased by priming.

This is the first study on JIA presenting data on HNL in relation to S100A8/A9 and MPO, as well as JADAS27. The study population was fairly small, as was the group of healthy children. Other shortcomings of our study were that samples were not collected pairwise, at high and low disease activity, and the heterogeneity of medical regimes, of categories of the disease among the participants and of the differing disease durations at inclusion. It was also a shortcoming that the level of JADAS27 overlapped between children without medical treatment and those with treatment. A strength was that several proteins from neutrophil granulocytes were included, all collected at the same occasions.

Much research has been conducted on S100A8/A9 and JIA, resulting in the use of S100A8/A9 as a biomarker, sufficient to monitor disease activity but most likely not to predict flares [32, 33]. Unlike S100A8/A9, the concentrations of MPO and HNL remain high in JIA patients even during use of immunosuppressive drugs. This could suggest that the current medical treatment does not manage to reverse the primed state of neutrophils, although it manages to decrease inflammatory activity. Our finding of increased serum concentrations of MPO and HNL also in a lower disease activity state, on medical treatment, most likely supports earlier findings that genes from neutrophils are upregulated even in low disease activity in JIA, as a sign of chronically dysregulated neutrophils [4, 8]. This has been shown to relate mainly to the impact of chronic inflammation but neutrophils in JIA were shown to exhibit considerable specificity in their transcriptomes [9].

## Conclusion

Neutrophil granulocytes in children with JIA are primed to release primary and secondary granule proteins, without relation to medical treatment whereas signs of increased turnover and sequestration of neutrophil granulocytes are reduced by treatment. Levels of neutrophil-originating proteins in serum most likely reflect underlying disease activities of JIA. We speculate that priming and activation of neutrophils contribute to the pathogenesis of JIA. We suggest that future studies investigate the process of neutrophil priming further, potentially laying the ground for novel ways to target the disease.

## Supplementary Information

**Additional file 1: Table 1**. Correlation analyses of HNL in serum related to clinical variables in 69 children with JIA.

**Additional file 2: Fig. 1**. Serum levels of human neutrophil lipocalin (HNL) in 16 healthy children and 69 children with juvenile idiopathic arthritis (JIA), presented in the different categories of the disease. The Kruskal-Wallis test was used.

**Additional file 3: Fig. 2**. Serum levels of S100A8/A9 in 16 healthy children and 69 children with juvenile idiopathic arthritis (JIA), presented in the different categories of the disease. The Kruskal-Wallis test was used.

## Data Availability

The datasets used and/or analysed during the current study are available from the corresponding author on reasonable request.

## Abbreviations

ELISA: Enzyme-linked immunosorbent assay
HNL: Human neutrophil lipocalin
IQR: Interquartile range
JADAS27: Juvenile arthritis disease activity score 27
JIA: Juvenile idiopathic arthritis
MMP-9: Matrix metalloproteinase-9
MPO: Myeloperoxidase
