# Supplementary material for: A cross-sectional cohort study of the activity and turnover of neutrophil granulocytes in juvenile idiopathic arthritis
Source: Pediatr Rheumatol Online J. 2021 Jun 30;19:102. doi: 10.1186/s12969-021-00600-7 (PMC8247147; doi:10.1186/s12969-021-00600-7)
Supplement: Supplementary file 1 — Additional file 1: Table 1. Correlation analyses of HNL in serum related to clinical variables in 69 children with JIA. [file 12969_2021_600_MOESM1_ESM.docx]

Additional Table 1. Correlation analyses of HNL in serum related to clinical variables in 69 children with JIA.

| Total cohort  n= 69 | | p-value* |
| --- | --- | --- |
| A comparison between concentration of HNL in serum and the following blood biomarkers: | | |
| B-leukocyte  B-neutrophil  S-CRP  S-MPO  S-S100A8/A9  S-MMP-9  JADAS27 | r = 0.41  r = 0.52  r = 0.43  r = 0.60  r = 0.50  r = 0.79  r = 0.02 | p < 0.001  p < 0.001  p < 0.001  p < 0.001  p = 0.001  p < 0.001  n.s. |

*Spearman’s rank correlation analysis.
