# Supplementary figures and images for: A cross-sectional cohort study of the activity and turnover of neutrophil granulocytes in juvenile idiopathic arthritis
Source: Pediatr Rheumatol Online J. 2021 Jun 30;19:102. doi: 10.1186/s12969-021-00600-7 (PMC8247147; doi:10.1186/s12969-021-00600-7)

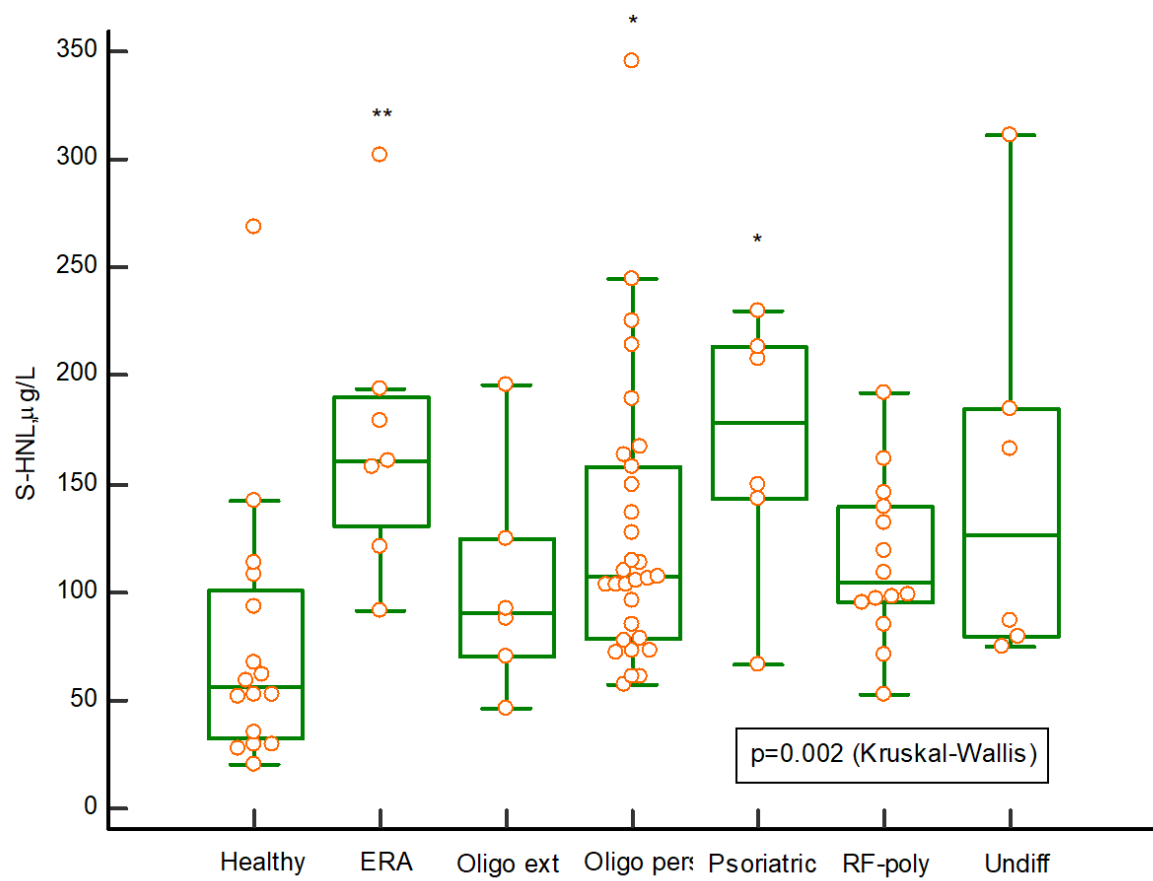

Supplement: Supplementary file 2 — Additional file 2: Fig. 1. Serum levels of human neutrophil lipocalin (HNL) in 16 healthy children and 69 children with juvenile idiopathic arthritis (JIA), presented in the different categories of the disease. The Kruskal-Wallis test was used. [file 12969_2021_600_MOESM2_ESM.pdf]

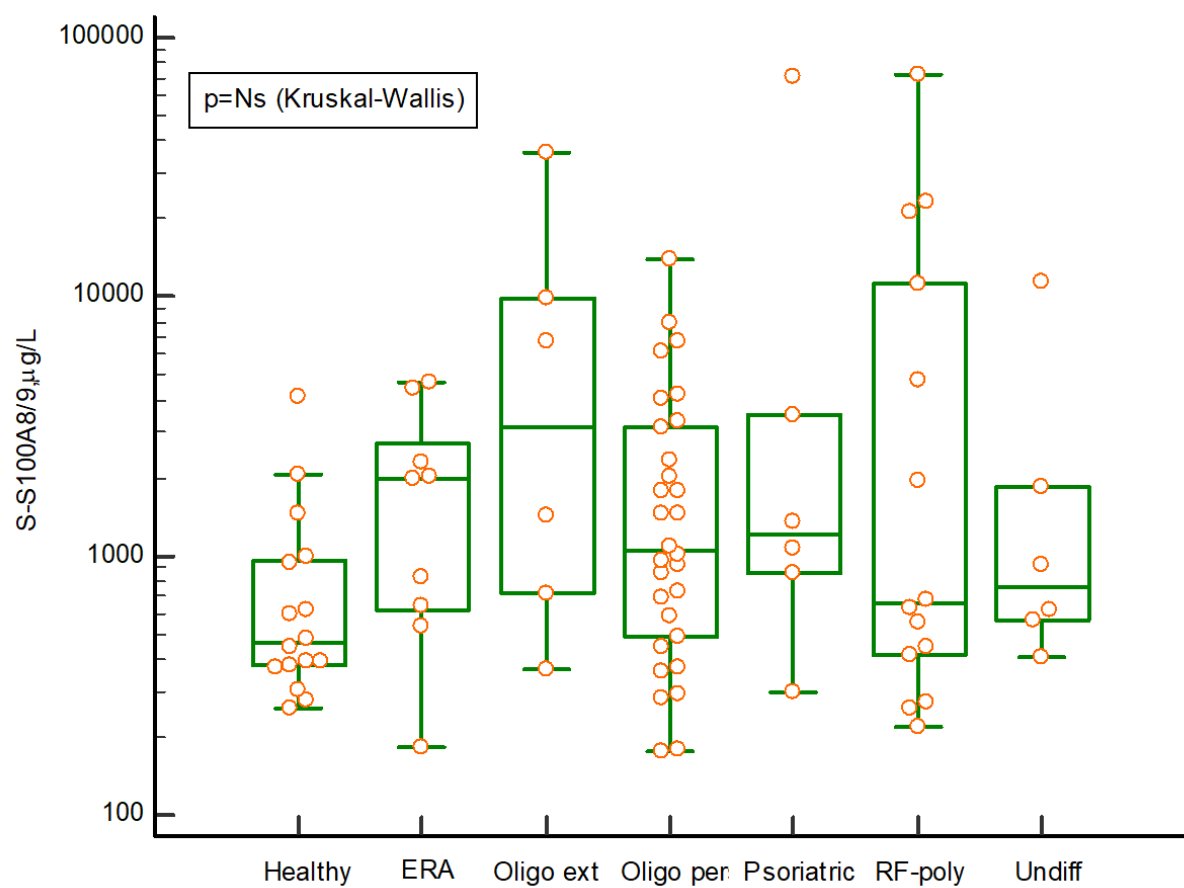

Supplement: Supplementary file 3 — Additional file 3: Fig. 2. Serum levels of S100A8/A9 in 16 healthy children and 69 children with juvenile idiopathic arthritis (JIA), presented in the different categories of the disease. The Kruskal-Wallis test was used. [file 12969_2021_600_MOESM3_ESM.pdf]
